# Predictors of Practice about Blood Borne Infections among International Medical and Paramedical Students Using the Health Belief Model

**Published:** 2019-09

**Authors:** Leonard Emmanuel MENSAH, Roya SADEGHI, Azar TOL, Abbas RAHIMI FOROUSHANI

**Affiliations:** 1. School of Public Health, International Campus, Tehran University of Medical Sciences, Tehran, Iran; 2. Department of Health Education & Promotion, School of Public Health, Tehran University of Medical Sciences, Tehran, Iran; 3. Department of Epidemiology and Biostatistics, School of Public Health, Tehran University of Medical Sciences, Tehran, Iran

## Dear Editor-in-Chief

Blood borne pathogens (BBP) are identified as pathogenic microorganisms, which exists in blood and can cause disease in people (1, 2). Despite this fact, blood borne infections can be avoided by applying general and specific measures of prevention and control (3). Medical and paramedical students are exposed to blood and other body fluids in the course of their practicum. Therefore, they are at risk of infection with blood borne viruses. The risk of infection for these students depends on the prevalence of disease in the patient population and the nature and frequency of exposures (4). Increase in the incidence of deadly infections due to greater exposure to microorganisms and viruses that causes blood-borne diseases has led the medical community to initiate efforts to prevent and limit exposure among health care workers including medical students, as a result, more than 1000 HCW contracted serious blood-borne diseases, such as Hepatitis B, C or HIV (5). Medical interns of Tehran University of Medical Sciences face a high risk of blood-borne infections through accidental blood exposures (6). Besides, vaccination coverage was an acceptable level but its effectiveness is not clear yet, the incidence rate of definite Cutaneous Blood Exposure Accident’s was 0.56 person annually(6).

This study aimed to assess the level of knowledge and predictors of practice about blood borne infections among international medical and paramedical students of TUMS using the Health Belief Model.

The ethical code of the study was IR.TUMS.SPH.REC.1396.2805. Informed consent was obtained from each participant before enrollment.

This cross-sectional study was conducted from Jun–Sep 2017. Most of the participants were male (69.7%), with mean age of 23.35, ±4.926 yr. The Mean score of knowledge and practice towards blood borne infections prevention among participants were 21.93(±6.39) and 9.19(±2.99) respectively.

Multiple variable analysis showed a significant association between marital status, education level, and knowledge. However, among the six constructs of HBM, only perceived barriers (*P*<0.017), and perceived self-efficacy (*P*<0.033) were significantly associated with preventive behaviors of blood borne infections (Table 1). The study concludes that adequate knowledge can lead to good practices to prevent blood-borne infections among medical and paramedical students of TUMS.

**Table 1:** Multiple variable analysis between practice and potential independent variables

***Variable***	***Univariate Analysis***		***Multiple Variable Analysis***	
	***β (95% CI)***	***P***	***β (95% CI)***	***P***
Knowledge	0.08 (0.06–0.09)	0.001	0.04(0.02–0.60)	0.001**
Perceived Susceptibility	0.20 (0.11–0.28)	0.001	−0.01(−0.10–0.08)	0.807
Perceived Severity	0.35 (0.26–0.45)	0.001	0.07(−0.05–0.19)	0.240
Perceived Barriers	0.41 (0.32–0.51)	0.001	0.148(0.03–0.27)	0.017*
Perceived benefits	0.36 (0.26–0.46)	0.001	0.03(−0.01–0.15)	0.654
Cue to Actions	0.32 (0.22–0.42)	0.001	0.07(0.04–0.18)	0.213
Perceived Self-Efficacy	0.40 (0.30–0.487)	0.001	0.13(0.01–0.25)	0.033*

* significant at *P*<0.05,

** significant at *P*<0.001

Thus, knowledge about blood-borne infections works through the constructs of HBM in predicting practice. Interventions such as ongoing training and awareness regarding infection preventions especially among undergraduate medical and paramedical students will be helpful in improving knowledge and thereby improve practice towards blood-borne infections. The greater the perceived barriers associated with blood-borne preventive behaviors, the less likely students will be to adopt such behaviors.

Perceived barrier associated with blood borne preventive behaviors was significant predictor of practice and students becoming more careful about standard precautions and making sure that they are infected with blood-borne diseases. Therefore, the research findings supported the inverse relationship between perceptions of the barrier and the practicing of certain blood-borne infections preventive behaviors. The predictive nature of HBM in relation to blood borne infections preventive health actions appears to have been greatly supported in this present research. Thus, perceived barriers and perceived self-efficacy are important predictors of the adoption of blood borne preventive behaviors among international medical and paramedical students tested in the study.
